# Factors That Influence HIV Risk among Hispanic Female Immigrants and Their Implications for HIV Prevention Interventions

**DOI:** 10.1155/2012/876381

**Published:** 2012-02-08

**Authors:** Amy M. Hernandez, William A. Zule, Rhonda S. Karg, Felicia A. Browne, Wendee M. Wechsberg

**Affiliations:** ^1^Substance Abuse Treatment Evaluations and Interventions Program, RTI International, 3040 Cornwallis Road, Research Triangle Park, NC 27709-2194, USA; ^2^Behavioral Health Epidemiology Program, RTI International, 3040 Cornwallis Road, Research Triangle Park, NC 27709-2194, USA

## Abstract

Hispanics are the fastest growing minority group in North Carolina with increasing incidence of HIV infection. Gender roles, cultural expectations, and acculturation of women may explain some of Hispanic women's risks. The perspectives of Hispanic female immigrants and community-based providers were sought to identify services they offer, understand HIV risk factors, and support the adaptation of a best-evidence HIV behavioural intervention for Hispanic women. Two sets of focus groups were conducted to explicate risks and the opportunities to reach women or couples and the feasibility to conduct HIV prevention in an acceptable manner. Salient findings were that Hispanic female immigrants lacked accurate HIV/AIDS and STI knowledge and that traditional gender roles shaped issues surrounding sexual behaviour and HIV risks, as well as condom use, partner communication, and multiple sexual partnerships. Intervention implications are discussed such as developing and adapting culturally appropriate HIV prevention interventions for Hispanics that address gender roles and partner communication.

## 1. Introduction

Hispanics are the fastest growing minority group in the United States and in the state of North Carolina [1–3]. As the Hispanic population has grown in North Carolina, so have the HIV cases among them. Eight percent of newly diagnosed HIV cases in North Carolina in 2008 were among Hispanics [4]. HIV prevalence among Hispanics in North Carolina was 3.7 times higher than among non-Hispanic whites, and among Hispanic women it was 4 times higher than among non-Hispanic white women [4]. While Hispanic women are disproportionately affected by HIV and sexually transmitted infections in the USA, paradoxically they tend to report lower levels of HIV risk behaviours than African-American and non-Hispanic white women [5, 6]. This raises questions regarding why these rates are higher.

One possible factor might be related to Hispanic cultural norms surrounding gender roles. Several of these norms may influence behaviour in ways that affect HIV risk and limit Hispanics' understanding of risk and the manner in which they communicate about it. The cultural norm for men known as “machismo” describes the role of the Hispanic man in his family and in society [7, 8]. While there are positive aspects to machismo, other elements of it such as the belief in male dominance and an emphasis on male sexual prowess are used by some men to justify sexual encounters outside of their primary relationship [9, 10]. The cultural norm for Hispanic women, “Marianismo,” strongly encourages virginity until marriage, being a devoted mother, and maintaining a submissive role with their male partners [7, 8]. The interaction between “machismo” and “Marianismo” among Hispanic men and women may lead to increased HIV risk by limiting a woman's ability to negotiate condom use or other safer sex behaviours. Newly immigrated women may be the least acculturated and hold less power in a relationship [11].

Another factor may be that recently immigrated Hispanic males in North Carolina, who often leave their families behind when they come to the USA, have been reported to engage in sex with commercial sex workers at relatively high rates [12, 13]. Another study developed an intervention for male Hispanic immigrants in rural North Carolina using a community-based participatory research approach with a local soccer league whose teams were composed almost entirely of Hispanic immigrants [14]. The intervention employed a peer navigator approach and resulted in reductions in risk behaviour [15].

Previous research suggests that interventions designed to reduce HIV risk among Hispanic immigrants must be sensitive to Hispanic cultural norms to be effective [7, 16, 17]. A systematic review of HIV prevention interventions for Hispanics in the USA concluded that single gender interventions (i.e., interventions for men only or women only) were more effective than mixed gender interventions in reducing HIV risk [18]. However, a qualitative study that conducted 13 focus groups with researchers, service providers, and heterosexual male and female Hispanics in Puerto Rico, the Dominican Republic, and Mexico concluded that for interventions to be effective they would need to include both males and females [16]. The same study identified important barriers that must be overcome to conduct successful couples-based interventions with Hispanics. Therefore, it is still unclear how to best reach Hispanics, and more formative research is required.

A recent qualitative study examined the intervention needs regarding sexual health of immigrated Hispanic women in North Carolina [19]. This study concluded that a culturally appropriate intervention should be designed for just these women. The intervention should utilize skill building and include simple sexual health knowledge. The study also recommended the intervention incorporate condom negotiation skills that recognize gender roles and power.

This paper incorporates findings from a two-phase formative inquiry involving Hispanic female immigrants and community-based providers who serve Hispanic women in Durham, Wake, and Orange counties in North Carolina. Our team had several goals in mind in conducting this study: to determine the services provided to Hispanic women; to identify known HIV risk behaviours and barriers to HIV prevention among Hispanic women; to assess their intervention needs; to help inform the adaptation of a woman-focused HIV prevention intervention, a CDC “best-evidence” HIV behavioural intervention [20]. This intervention, the Women's CoOp [21], has been adapted by our team for multiple at-risk women domestically and globally as HIV infection has affected many disempowered women [22]. While some interventions have been based on empowering women [23, 24] or specifically working with Hispanics [25–27] or Hispanic youth [28, 29], few if any have been developed for adult Hispanic females that include a personal plan to evaluate and address risky behaviors, including substance use, in a culturally appropriate manner. The Women's CoOp incorporates personalized HIV risk assessments along with rapid HIV testing, substance abuse knowledge, sexual risk, and education about STIs and violence prevention with harm reduction methods with culturally congruent peers and role playing. It combines an individualized appraisal of several types of risky behavior, including partner risk with intervention sessions to improve communication skills. The Women's CoOp is a portable woman-focused intervention that works by empowering women to increase their concrete problem-solving skills, reduce their sexual risk behaviours, and decrease their risk of violence and victimisation and can be conducted in health clinics and in doctor's offices. However, at the core of each adaptation is a process of working with targeted community members and their social supports to best determine the adaptation process.

Family medical providers can now play an important role with rapid testing in addressing HIV prevention with Hispanics and have the potential to offer linkages to other care or offer STI treatment [30, 31]. Since Hispanics are more likely than other groups to delay the onset of medical treatment once diagnosed with HIV [32, 33], early testing, treatment, and prevention interventions for Hispanics must be provided by family physicians to reduce HIV transmissions, assist those with the virus, and improve health outcomes. Though the field of family medicine has demonstrated HIV prevention and care programs can be incorporated into clinical practices [34–36], there is little knowledge regarding culturally competent clinical strategies geared towards Hispanics or Hispanic female immigrants [37]. The Hispanic Women's Health Project is one step toward addressing the gap in knowledge, in the field of family medicine, related to HIV prevention interventions for Hispanic women if an intervention could be adapted and placed in easy-access settings.

## 2. Materials and Methods

The Hispanic Women's Health Project was a qualitative study to assess the potential need and the feasibility of a community-based HIV prevention intervention for newly immigrated Hispanic women in North Carolina. The study was conducted in two phases by two investigators over several years and included focus groups with immigrated Hispanic women and community-based health care providers who served them. The first phase of the adaptation process was conducted by one investigator in September and October 2006 and entailed six focus groups with 40 at-risk Hispanic women and providers who delivered health services to newly immigrated Hispanics in Durham and Chapel Hill, NC, USA. The second phase of the adaptation took place from January 2009 to March 2010 and involved another investigator who conducted three focus groups in June 2009 with 26 service providers who worked directly with Hispanic women and represented several community-based agencies.

The first phase of the project received full approval from RTI International's Institutional Review Board (IRB). phase two of the project received an exemption from RTI International's IRB because focus groups were with service providers who were considered consultants.

### 2.1. Recruitment

Organisations in Durham, Wake, and Orange counties were first contacted to ensure the support of influential and established community organisations and to serve as venues for both phases of this study. A community-based agency in Durham County and another in Orange County hosted the phase-one focus groups. For phase two, one organisation from each of the major counties agreed to host a focus group.

For phase one, participants were recruited through both street outreach and announcements at community organisations for Hispanics. Staff conducting the street outreach screened volunteers to determine if they were eligible to participate in the focus groups. Because the target population for an HIV prevention intervention would be Hispanic women who were at risk for HIV, eligibility criteria included having two or more dating or sexual experiences with men in the past 12 months. Because HIV prevention messages are relatively common in the USA, more recently immigrated Hispanic women were considered to be more at-risk for HIV than those who had lived in the USA for a longer period of time. Therefore, other eligibility criteria included being a female Hispanic immigrant, being at least 18 years old, speaking Spanish as their primary language, and having had two or more dating or sexual experiences with men in the past 12 months. Of the 63 women who were screened for the study, 51 were eligible and invited to participate in the focus groups. Reasons for ineligibility included: an unwillingness to answer questions on the screening questionnaire (*n* = 7) and being less than 18 years old (*n* = 5). Of the 51 Hispanic women who were scheduled for the focus groups, 11 did not attend the groups for unknown reasons.

For phase two, potential focus group participants were identified through several methods: attendance at a local Hispanic seminar, local alliances, or groups focused on assisting or empowering Hispanic women, telephone books, community resource guides, internet searches, and an established Community Advisory Board (CAB) for the Women's CoOp studies and other studies. Of the 93 service providers contacted, 33 (35%) did not respond to our telephone or email contacts, 20 (22%) declined to participate, 12 (13%) were not eligible, and 28 (30%) were eligible and agreed to participate. Of the 28 who were eligible and agreed to participate, 26 attended the focus groups.

### 2.2. Protocol

The focus group discussions for phase one and phase two were held at three local organisations in the evening and lasted approximately two hours. Most of the focus groups were held at nonprofit organisations for Hispanics in Durham, Wake, and Orange County, and one was held at a county health department. Participants from both phases of the project were first asked to describe the Hispanic women in the area, the available services or programs offered to them, and the issues Hispanic women face. Second, participants were asked to review the Women's CoOp intervention cue cards and provide input on how they could be adapted for use with Hispanic women. Third, participants were asked their opinions about how best to conduct an HIV prevention intervention with local Hispanic women. In order to gain knowledge about their understanding of the Hispanic population they served, their perspectives as members of their respective organisations were sought. All the questions asked were related directly to their work as staff members from community-based organisations who work with Hispanic females. Participants were instructed to talk about their experiences, in some cases as immigrant Hispanic women and as service providers, but not talk personally about their own behaviour or experiences outside of their service provider perspective. Focus group participants in phase one received a $30 stipend and dinner for their participation. They were also reimbursed up to $20 for transportation if they paid for a taxi, bus, or other transportation to and from the focus groups. Focus group participants from phase two received a $25 stipend for their participation, in addition to dinner.

The phase-one focus groups were led by two Hispanic women indigenous to the Hispanic community who were fluent in English and Spanish and had experience conducting community-based health research and interventions with Hispanic women. Both of the leaders and the investigator of phase one took notes during the participants' discussions. The phase-two focus groups were led by the investigator of the second phase, who is fluent in English and Spanish, with assistance and note taking from a research assistant, who is also fluent in both languages. While focus groups were primarily conducted in English, participants were encouraged to speak in the language they felt most comfortable. As a result, some of these discussions were conducted in Spanish. To supplement note taking, all discussions were audio-taped. After the group discussions, the note taker transcribed the audio tapes verbatim.

### 2.3. Qualitative Coding and Analysis

A number of steps were taken to ensure quality of the data. After each focus group in phase one, the discussion leader, note taker, and investigator compared their notes to verify completeness and to correct any commissions or omissions. These notes were then consolidated onto one set of transcripts for each focus group and were reviewed again for accuracy by the team. Next the team met to determine themes that emerged from the six focus groups. The focus group discussions were not audio recorded due to concerns that doing so could interfere with participation. Therefore, verbatim transcripts for this phase of the study were not used for the analyses.

Focus group discussions from phase two were recorded, allowing the note taker and the investigator to compare the audio files to the transcriptions for completeness and accuracy. For phase two, the final verbatim transcripts were imported into ATLAS.ti (Version 5.6.1), a specialized qualitative software. A set of qualitative descriptive code definitions and coding procedures were drafted by two coders and refined through an iterative process. Code definitions were based on the focus group topics and major themes of the study. A formal assessment of intercoder reliability was independently performed by both coders with a subset of text consisting of 10% of the full analysis sample [38–40]. Coders carried out additional intercoder reliability exercises until they achieved 80% reliability. Coders then independently coded all data during the formal coding phase. Coders met to discuss ambiguous text and reconcile discrepancies before the coding was considered complete. The first author conducted the data analyses for the findings reported in this publication which entailed the query and extraction of relevant passages that have been synthesized and summarized below.

### 2.4. Participants

For phase one, the age range of the 40 Hispanic female focus group participants was 18 to 41 years, with an average age of 29. All of the women were first-generation immigrants, living in the United States for two to seven years (average 5.5 years). The female participants from phase one stated they migrated from Mexico (*n* = 30), Honduras (*n* = 5), and Guatemala (*n* = 5). The majority of the women (75%) were married or living in a free union with a partner; primarily the younger women (18 to 20 years old) were single.

In phase one, about 20% of the sample was proficient in English. Nearly half of the sample had completed six to nine years of education, another third had at least some high school education, and one woman had earned an advanced degree. All had been primarily educated in their native country, but approximately one-third had also received some education in the United States. Most of the women worked outside the home, mainly in unskilled labour positions. Although women were not asked whether they were documented or undocumented immigrants because they were fearful to share this information during the screening process, many made comments during the group discussions that suggested they were undocumented.

For phase two, the 26 service provider focus group participants ranged in age from 25 to 61, with 22 women and 4 men. More than half were identified as Hispanic (*n* = 15) while smaller numbers were identified as non-Hispanic White (*n* = 8) or non-Hispanic Black/African American (*n* = 3). Participants had been at their current positions from six months to 15 years.

## 3. Results

In both phases, the majority of participants reported that most Hispanic women in North Carolina were newly immigrated, with 65% from phase two stating that almost all were in the USA illegally and without proper documentation. While 20% of the phase-one participants reported English proficiency, a total of 73% of the phase-two participants who provided services to Hispanic women stated their clients predominantly spoke Spanish. According to several service provider participants in both phases, the vast majority of their Hispanic clients were from Mexico. Smaller percentages were from Central American countries, including El Salvador, Guatemala, Honduras, and Panama. The majority of participants stated almost all of their Hispanic clients were poor and had low incomes. According to many service providers in phase two, most of their clients had very little formal education. Although some of their clients were able to read and write at a third-grade level, many of them were functionally illiterate. In all of the phase-two focus groups, the majority of respondents stated their Hispanic female clients were in relationships with Hispanic men. The partners of the Hispanic female clients, according to most service providers, were most often Hispanic males.

### 3.1. Types of Services Provided to Hispanic Women

Community-based service providers who participated in phase-one and phase-two focus groups provided a range of services to their Hispanic clients who resided in Durham, Wake, or Orange County in North Carolina. The organisations that they worked for included Hispanic community centres that offered education, youth, and health promotion programs, county health departments, and local community health centres. Some participants provided services related to reproductive health, sex education, and HIV/STI testing and counselling. Other organisations provided behavioural health and substance use counselling. Participants also delivered mental health services for Hispanics experiencing trauma, violence, crime, domestic violence, and rape or sexual assault.

### 3.2. Factors That Influence HIV Risk among Hispanic Female Immigrants in the Triangle

#### 3.2.1. Lack of HIV/AIDS and STI Knowledge

According to service provider participants from phase one and phase two, their Hispanic clients hold misconceptions and believe in a variety of myths regarding STIs, such as contracting HIV by simply looking or touching a person with the virus and others listed below, see Table 1. One particular service provider spoke about a myth related to mosquitoes and explained “Because when you are talking about the mosquitoes, some people say it's true that mosquito can transmit the HIV.” This provider described how some Hispanic clients falsely believe this myth. Providers also reported that many of their Hispanic clients did not understand the difference between HIV and AIDS as reflected in the statement below from a service provider

We, in our culture we don't think about HIV. We think about AIDS. Oh he has AIDS. She has AIDS. We don't say HIV. Not until you know and learn that HIV causes AIDS. So we say AIDS, AIDS, AIDS … AIDS, she has AIDS, he has AIDS. You know, we don't say she has HIV…

Focus group participants from both phases noted that Hispanic women are at risk of HIV and other STIs. The majority of Hispanic participants from phase one reflected Hispanic women's lack of knowledge of safer sex practices. In half of the phase-one groups, few Hispanic women were knowledgeable about the proper application of a male condom. Participants in two-thirds of the focus groups were also unfamiliar with female condoms and their usage. Several providers and Hispanic females from both phases described how Hispanics were not familiar with the symptoms, the routes of transmission, or their personal risk factors for STIs and HIV. When discussing the symptoms of HIV, a group respondent who provided HIV-related services articulated a common misunderstanding of the symptoms and fear among some Hispanic clients:

… all these symptoms could be symptoms of any number of things. And so people come in because they have a rash and they think they have HIV… And so when people ask about symptoms we were told to say that they were very much like the symptoms of a cold or flu. … but we do not spend a lot of time on it because you know I have a lot of people coming in to test who just have you know an ache, or you know, not anything, nothing.

Yeah, it's scary for them, every time they get an itch.

Most Hispanic females from phase one demonstrated the need for HIV/AIDS education during focus groups. During phase two, when a service provider asked Hispanic women how to handle the diagnosis of HIV, the women often explained false information about their own risks in relation to the ways in which HIV can be transmitted to their spouses.

What happens if you know you have HIV? [Hispanic women respond by saying,] “I do not worry; I take a shower every day. My husband takes a shower every day.” So they think that they are clean, that he is clean, because they shower every day.

[Everyone agrees].

Numerous group participants reported that Hispanics shared several misperceptions about STIs and HIV, including those described above.

#### 3.2.2. The Impact of Traditional Gender Roles on Sexual Behaviour

Hispanic women and service providers often described Hispanic women mostly in terms of traditional gender roles. The majority of service providers and Hispanic females stated their male partners were Hispanic men. In both phases of the study, they were depicted as often being dependent upon and subservient to their Hispanic male partners. The primary roles of Hispanic men were often defined as being the decision makers and monetary contributors of their families. Female focus group participants described how the cultural tradition of “machismo” usually dictates that Hispanic men make all the decisions for their wives and families. Hispanic women, on the other hand, were said by a number of participants to be the caretakers of the children and responsible for household duties, which reinforced their “Marianismo” role

“… like if they are married, you know the husband is the one who's working and she is always sitting at home, you know watching soap operas…and taking care of the kids …”

“Yes, it's they have babies and babies and babies, cause this is their way. They [Hispanic men] can have their lady all day busy and at home. This is why we have the Latinas here with the big families in the United States.”

“…  and the women have to stay at home, cause she's the woman, you know, she's not like we are equal.”

Hispanics were frequently reported by providers and the women themselves to have clear inequalities between men's and women's expected behaviours in the USA that often reflected “Marianismo” and “machismo” gender roles. Some Hispanic women stated that their Hispanic male partners made family decisions, exhibiting “macho” behaviour, now that they reside in the USA when extended families live outside of the country. They disclosed that this was partially because their families could not intervene from a distance on their behalf. Hispanic women said that the needs of the family come first before their own and they were expected to “be a good mother” and to “scarifice your needs” in their culturally encouraged, “Marianismo,” gender-specific role. Participants from both phases described Hispanic gender roles mostly based on traditional concepts that included a self-sacrificing, subservient Hispanic female homemaker, “Marianismo”, role and a dominating, Hispanic male family decision maker, “machismo” role.

#### 3.2.3. Gender Roles, Condom Use, and Partner Communication

According to many participants in the Hispanic women and provider groups, Hispanic women tended to be submissive about sexual behaviour. Because discussing sexuality was culturally prohibited or forbidden, Hispanic women were often reported to have experienced uneasiness and trepidation when discussing the topic, as this quote from a Hispanic female provider illustrates

Personally, you talking here about relationships, I think communication is key. To me, Latinas have come from a different background. We tend to be more, we were taught to be more submissive, more private, not to talk about this openly with our partners, about sexuality or that [safe sex]. So really, you have to incorporate some of our cultural taboos, fears, there is a lot of fear, to be able to talk about these things.

Some Hispanic female respondents also described how conversing about sex was culturally inappropriate and “a taboo” for Hispanics

Using the word sexuality will bring a lot of taboo, especially, depending on the age range. If you are talking to teenagers it will be okay, but then, you know, it's a taboo all the time to talk about sex. How do I talk about sex? You know, I'm married, I don't know anything else. I've only had one partner.

…  talking about sexuality, that's a code word too, they are going to say “shut-down”, “done.”

In two-thirds of the phase-one groups, the participants discussed how initiating a discussion related to sex would be considered culturally improper. They discussed “the culture of obedience,” which represented the “Marianismo” role, as a common influence on many aspects of Hispanic women's lives including their sexual behaviours. This culturally common, gender-biased role was said by Hispanic women to involve submission to men's decisions, desires, and power. A participant from phase two stated “… we don't talk about it [sex], because it's like a cultural thing where the guy makes the decisions.” A frequent response from Hispanic women was “My husband does not let me.” Hispanic male partners, as most women explained, are often the “macho” patriarchs and must give permission for their female partners to pursue certain interests, even in the bedroom.

According to several respondents, Hispanic women allowed their decisions about condom use to be made by their male sexual partners who were said to most likely be Hispanic themselves. Some participants in both provider and Hispanic female focus groups stated that Hispanic men disliked condoms and did not use them

“Yeah, because they really don't like to use the condoms …  the Latino men. I don't know why. I really don't know why, but they don't like to use the condom.”

Some providers stated their organisations provided safe sex workshops for Hispanic women. Some Hispanic female participants admitted to needing training in proper condom use and condom negotiation. Respondents from one focus group with Hispanic women stated they did not have a problem using a condom if their partner suggested it. However, a few providers briefly discussed the arguments that occurred between partners when women obtained condoms during these workshops

I am reminded of another barrier, it's a big one I think, is that Latino culture. Because I hear it from many ladies, when they come in here and somebody do workshop about the um, the safe sex, safer sex, or prevention, or protection sex. If she go back to the home and her husband want to see one condom, it's a big problem for that lady. She be in trouble with her partner because he says, “Why you carry a condom, explain it to me?” [Then the Hispanic woman would say,] “No, I went to [name of organisation], somebody talked about prevention and safer sex.” They do not understand the situation and it is big, big problem. I think that Latino men they have to educate about different situations.

[All AGREE: say yeah, that's true.].

Many respondents from both the provider and Hispanic female groups shared that Hispanic women were unsure of how to bring up the topic of sexuality with their partner and often felt embarrassed about it. Various women stated they “don't know how to talk about safe sex” with their partners. Several group participants stated Hispanic females felt discomfort, shame, or fear about talking to their partners about sex and negotiating condom use as described below.

In my experience …  some ladies, for example I am talking about safer sex, about how they can condom use negotiation with their partners. Sometimes I explain to them to tell their partner or boyfriend, “If you go outside, every weekend, he had to carry one condom or two condoms with him for any situations.” But the lady say, “No. Why I can tell my husband, okay take two condoms? No, no, I can't.”

Latino woman thinks, many Latino women thinks things like that. But I explain to her that if you don't tell your husband or your partner, he can do whatever, and the consequences for you are bad.

When Hispanic female participants were asked why some Hispanic women who have safe sex knowledge still engage in unprotected sex acts, they explained their fears that a man will think they are too experienced with sex: “Men will think that you are prostitute if you know a lot about sex.” Hispanic men who exhibit “machismo” typically had power over the sexual behaviours in their relationships, a number of respondents from the two phases of the study explained. According to respondents from two of the phase two focus groups, when Hispanic women were able to overcome their fears and attempted to negotiate condom use, Hispanic men may have accused them of having other sexual partners. Some participants explained, as the quote below illustrates, this led to compliance by the Hispanic women because of their children or for financial reasons

I think if they use it, if the woman decides to use it because of the knowledge she gained or got from someone. And comes home and says, well I am going to try and use a condom. The guy would be like, “Why? Are you sleeping with somebody?” You know, usually because of the role of the Machismo. You know, it's the guy who controls the, sexual acts in the relationship. And because they say, “Because of my children, I can't do nothing. I can't fight because I have two kids,” and then they do it, I hear it every day, so it's common. Sometimes, women you know, they are single mom's here. They know a guy, who is married in [Latin American Country], and they still get together here even though he is going to go back, you know. But still for financial situations, you know she has to find somebody to support her. You know we see that more.

When some of the Hispanic females that received services were described by phase two participants, their disempowered role was usually mentioned in relation to their husband's extramarital and substance use behaviours. One particular Hispanic male participant expressed the interconnected nature of gender roles, alcohol venues, condom use, and the misunderstanding of STI risks in relation to different sexual partners

But the men are at the bars and they be with another woman and they come home at like 4 in the morning, and nothing happened, because usually if she [main partner] stay home, then she [main partner] is safe, so why do I need to use to a condom, she [main partner] is safe, that's how I see it.

#### 3.2.4. Multiple Sexual Partnerships

Many provider and Hispanic female group respondents described that Hispanic men were involved with multiple sex partners. Some Hispanic female participants from phase one shared their fears about their male partners having other female partners if they did not “always please a man.” During phase one, Hispanic women in three-quarters of the groups stated that extradyadic sex was not acceptable but tolerated. They also stated that it was fairly common for men to have extradyadic relationships, especially those who are in the USA away from their wives. Two of the provider groups from phase two detailed the activities of the Hispanic males who were said to have attended local bars, while others had travelled to other states or countries and were involved with different sexual partners or sex workers. At times, the providers explained, these sexual encounters led some Hispanic men to seek STI testing and treatment. Participants from the phase-one Hispanic women's focus groups shared that Hispanic men, because of the role of “machismo,” tend to believe they are invulnerable to HIV and other STIs and would refuse to use condoms. Some providers described how Hispanic males' multiple partners led to their main partners contracting STIs or HIV

Respondent 1: … I have seen the other side too, the wife, where it's the man the one that leaves and he comes back like nothing, and the woman comes to the clinic and is full of diseases, and they keep coming because they can treat her, but he keeps going, so it's pretty sad.

Respondent 2: And usually what she says is that it happened like you know, with my friend. He went to [name of town], then came back, got sick, but she never thought that it was going to be HIV, you know. And it happens, but now they divorce, but it doesn't have to be in other countries, it can be within the states.

### 3.3. Intervention Implications

#### 3.3.1. Influence of Education, Illiteracy, and Income on Intervention Development

Because of the low literacy levels and little formal education among many Hispanic clients, provider participants from phase one and phase two explained the need for the use of simple language and much repetition when working with them. The overwhelming majority of focus group participants stated the HIV prevention intervention must be in Spanish. Since nearly all of their Hispanic clients spoke only Spanish, any intervention for Hispanic women must be in Spanish. A great deal of visual material for the intervention information, such as the use of pictures or videos, was stressed by groups from both phases as essential with this population. Interventions should strive to limit the amount of information presented in a written format. Hispanics could be allotted time to share personal experiences that relate to the intervention topics since speaking about their lives would be a good approach to working with those that cannot read or write. Furthermore, it was suggested that interventionists share stories or read testimonials from other Hispanics who had relevant experiences about the salient intervention themes.

#### 3.3.2. Fostering HIV/AIDS and STI Knowledge among Hispanic Women

Hispanic females and service providers from phase one and phase two stated that increasing knowledge can occur during an intervention that is culturally sensitive to Hispanics if it includes interactive sessions focused on skill building. A few provider participants from phase two said “Start with the assumption that the audience knows nothing about this, and build from there…” Simple explanations on specific male and female anatomy may aid in educational sessions about routes of transmission. Repetition of these routes was discussed as being vital to intervention adaptations for Hispanics, along with participatory activities that foster discussions and active participation. Clear and direct explanations with several pictures that illustrate the definitions of and differences between HIV and AIDS were highly recommended by participants in both phases of the study. Hispanic female participants from phase one expressed enthusiastic support for developing and implementing a community-based group intervention that educates women about HIV, teaches skills for condom use and communication with their partners, and provides access to HIV testing and referrals to services.

#### 3.3.3. Gender Roles and Hispanic Sexual Behaviour

There are several implications for an HIV prevention intervention when considering Hispanic gender roles and the cultural influences on sex risks. Because of enmeshed Hispanic relationships, any HIV prevention intervention with this ethnic group may need to be more family focused, rather than individually focused, as this quote demonstrated.

We do not talk about me, me, me, and we talk about how our families are going to be affected. There is no I, there is a WE, we as a family, we as a community, we do not talk about I, I, I.

Service provider group members from phase one and phase two highlighted the need for an intervention to stress a more family-oriented view on health, which should demonstrate how topics can assist Hispanic families. Incorporating activities for the entire family was suggested by a few focus groups. Both Hispanic males and females may need to be involved in an intervention to address their interdependent gender roles. Most Hispanic females believed their major role as women was to take care of their husbands and children, according to several participants from phase one and phase two. Therefore, an intervention needs to foster this caretaking, “Marianismo”, role while emphasizing the positive impact HIV prevention will have on their families as a whole.

Many focus group participants from both study phases recommended increasing partner communication skills, including how to discuss sexuality, STIs, and condom use. Intervention strategies can incorporate Hispanic gender roles when addressing communication. Hispanic women respondents suggested teaching women to communicate messages to their male partners that appeal to “machismo,” such as “You want to be a better lover, learn to have safe sex.” They also recommended specific methods for addressing cultural norms about condom use, such as “If a woman asks a man to wear a condom, she is an intelligent woman not a whore.” Hispanic men's infidelity, encounters with sex workers, and unsafe sexual practices also need to be approached in a culturally appropriate manner since numerous participants expressed their difficulties with discussing these issues with their partners.

## 4. Conclusions

The perspectives of Hispanic female immigrants and community-based organisation staff were sought to identify services they offer to Hispanic women, understand HIV risk factors, and support the possible adaptation of the Women's CoOp HIV prevention intervention for Hispanic women. Many participants explained the limited or incorrect knowledge that Hispanics had about HIV/AIDS and other STI risk factors and transmission. The overwhelming influence of traditional gender roles among Hispanics was depicted by several Hispanic women and provider respondents, especially in relation to sexual risk factors for STIs such as multiple sex partners and sex trading. 

Findings from Hispanic women and service providers in North Carolina's Durham, Wake, and Orange counties underscore the need for HIV prevention interventions for Hispanic women that address specific cultural aspects and gender role expectations. Recent studies have emphasized the need for HIV prevention interventions to be culturally sensitive and appropriate when working with Hispanics [7, 16, 17], and this study supports these findings. Hispanic culture and gender roles, such as “machismo” and “Marianismo,” must be included in interventions with Hispanic women. Focusing on risk reduction for family well-being was highlighted in both phases of this study as well as others [8, 10]. Since Hispanic men were reported by community-based service providers to be responsible for and felt concerned about the well-being of their families, prevention strategies might include suggestions for demonstrating health-promoting behaviours as a sign of their family accountability. For example, being responsible can be reflected in Hispanic males' acts of responsibility through safe sex practices. In terms of Hispanic women, interviewees from both phases of the study described how they were centred on the well-being of their family members and suggested specific areas, mostly involving their marital relationships, to target during an intervention. If an intervention takes a family wellness approach, then the Women's CoOp will need to expand on improving couple relations, but also address both partners using alcohol and its effect on violence and developing problem-solving and communication skills, it is likely to be beneficial to the Hispanic population. Similar to other studies that stated both Hispanic women and men should be involved for successful HIV prevention interventions with this population [16, 41], focus group participants from phase one and phase two recommended that Hispanic women and their male partners participate in some type of culturally congruent intervention.

A major finding of the Hispanic Women's Health Project involved the traditional gender roles of Hispanics which included imbalances in power and shaped the issues surrounding HIV risk. Not only are traditional gender roles, like “machismo” and “Marianismo,” revealed among the Hispanics described within this publication dichotomous in nature, with Hispanic men serving as the decision makers and Hispanic women as the family caretakers, but these well engrained roles permeated sexual behaviour and partner communication [10, 42]. With men controlling sexual behaviour, specific risky behaviours, such as Hispanic men engaging in unsafe sex with multiple partners and purchasing sex from commercial sex workers, may have further increased the spread of STIs among the Hispanic population, thus the need for safe sex practices within couples-based prevention efforts. Future development of prevention interventions may need to include formative focus groups with men to learn how to tailor efforts with Hispanic men and address their involvement with main partners and other risky behaviours such as substance abuse.

Based on our findings, it appears that recently immigrated women may benefit from a woman-focused intervention, such as the Women's CoOp, that is adapted for Hispanic women which strives to educate and empower women. Our findings suggest that it may also be important to work with Hispanic males since they seem to be an important source of women's HIV risk. The Women's CoOp synthesizes substance use, HIV prevention, and harm reduction skills for women in a culturally appropriate intervention that can benefit Hispanic women as well as their male partners and can be placed in a health department or with medical providers within a rapid testing framework. Advances currently under trial for a Men's CoOp and Couples CoOp may also show promise for possible adaptations to Hispanic couples. Therefore, it may be more appropriate to work with Hispanic couples and address gender roles, expectations, and communication within the family while also focusing on HIV status, sexuality, and risk reduction in a loving fashion. Power imbalances may be a barrier that will need an honest appraisal within relationships so that men would not feel threatened and women would not be fearful to change. Additionally, future studies with Hispanics need to appropriately address both cultural and linguistic considerations when developing HIV and STI prevention strategies with Hispanic immigrants. Further development and adaptations of Hispanic couples-based intervention protocols will need to incorporate simple, skill-building sessions, highlighting the positive aspects of gender roles, building on the strengths of relationships and utilizing culturally competent and greater visual materials.

## Figures and Tables

**Table 1:** Factors that influence HIV risk among hispanic female immigrants in Durham, Wake, and Orange counties, NC, USA.

Factor	Illustrative quotation
Lack of HIV and AIDS knowledge	“Also, for the Latino culture when I invite somebody, come in, come in to testing for HIV. They say no, I don't have the AIDS. No I don't have AIDS. They think it's the same, HIV and AIDS.”
	“… if I know you have AIDS, you know, everybody thinks that if I look at you, I am going to get it, you know. If I touch you I am going to get it, you know?”
Traditional gender roles and condom use	“Yeah, you hear it all the time, “Why don't you use a condom?” [and the Hispanic woman would say] “Because my husband don't like it.”
	“… the women who are most at risk [are] … the ones who know that their husband has had sex with prostitutes on the weekend, or goes out drinking, and they know that he is having sex with her and they just, keep their mouth closed, because talking is riskier and they don't have a way of getting out of it anyway …”
Multiple sexual partnerships and unprotected sex	“And I find that the men who have families in their home town will want to come in and get tested before they go home. So you know, they'll want the results because they are going back to [Latin American Country] in a couple of weeks and they will want to get the test done, so, of course that means that they have been doing, they have been having unprotected sex here, but they want to be fine for their wives when they go home, or make sure that they are.”
